# Operating organic light-emitting diodes imaged by super-resolution spectroscopy

**DOI:** 10.1038/ncomms11691

**Published:** 2016-06-21

**Authors:** John T. King, Steve Granick

**Affiliations:** 1 Department of Materials Science and Engineering, University of Illinois, Urbana, Illinois 61801, USA; 2 IBS Center for Soft and Living Matter, UNIST, Ulsan 689-798, South Korea

## Abstract

Super-resolution stimulated emission depletion (STED) microscopy is adapted here for materials characterization that would not otherwise be possible. With the example of organic light-emitting diodes (OLEDs), spectral imaging with pixel-by-pixel wavelength discrimination allows us to resolve local-chain environment encoded in the spectral response of the semiconducting polymer, and correlate chain packing with local electroluminescence by using externally applied current as the excitation source. We observe nanoscopic defects that would be unresolvable by traditional microscopy. They are revealed in electroluminescence maps in operating OLEDs with 50 nm spatial resolution. We find that brightest emission comes from regions with more densely packed chains. Conventional microscopy of an operating OLED would lack the resolution needed to discriminate these features, while traditional methods to resolve nanoscale features generally cannot be performed when the device is operating. This points the way towards real-time analysis of materials design principles in devices as they actually operate.

## Abstract (web summary)

There is a need to characterize devices during operation in real-time and at nanoscopic length scales. Here, King *et al*. perform electroluminescence-STED imaging with a polymer based light-emitting diode, revealing nanoscopic defects that would be unresolvable with traditional optical microscopy.

## Graphical abstract

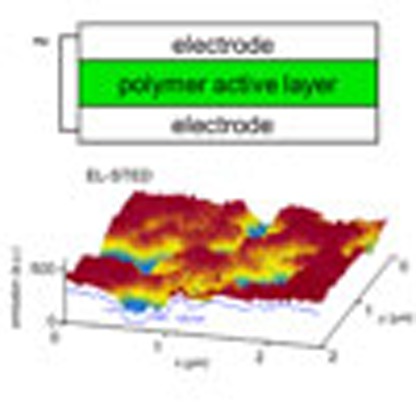

The ongoing revolution in diffraction-unlimited optical microscopy has led to a new arsenal of imaging techniques^1^,^2^,^3^,^4^, whose promise was recognized with a Nobel Prize in 2014 (refs 1, 5, 6). Importing these work-horse methods into materials science has been slow due to the lack of sophisticated labelling procedures which have been so successful in biology^1^,^2^,^7^. A particular area of potential application is operating functional devices. About the macroscopic behaviour of organic light-emitting diodes much is known and even brought to market commercially, and much also is known from post-mortem characterization at the nanometre scale^8^,^9^,^10^,^11^, but the scientific issues of achieving optimal macroscopic performance demand understanding mechanisms at all length scales.

While it is true that several current methods are capable of measuring ultrafast carrier dynamics with high-spatial resolution^12^,^13^,^14^, they do not apply during actual operation of the device. Other methods such as atomic force microscopy offer exquisite sensitivity of surface morphology but not of the interior of films, which is a limitation when dealing with electro-optical devices the bulk of whose signal is believed to come from beneath the film surface. Thus, there is an unmet need to characterize devices during actual operation and at nanoscopic length scales that reach below the film surface. The underlying polymer physics, known in principle, shows that the useful functions depend on the complex chain morphology in spin-cast films^15^,^16^,^17^,^18^,^19^, the relevant length scales of chain organization lying below the diffraction limit and hence below the capability to characterize by conventional optical methods.

Here we explore adaptions of STED microscopy that allow for real-time assessment of device function in operating devices. Emission is stimulated by electrical current^20^,^21^,^22^,^23^,^24^, thus circumventing need for fluorescence labelling, and is intimately linked to the underlying film morphology typically characterized at the mesoscale and below rather than the intermediate regimes addressed here.

Two specific adaptations of the usual STED microscopy are critical to accomplish this study: (1) electroluminescence-STED (EL-STED) to image electroluminescence maps with roughly 50-nm resolution in the *x–y* plane, and (2) STED-spectral imaging to map local-chain morphology in the emissive layer through differences in spectral response. Using these new techniques concurrently allows one to directly correlate nanoscopic defects with local-chain packing.

## Results

### Device architecture

**Figure 1:**
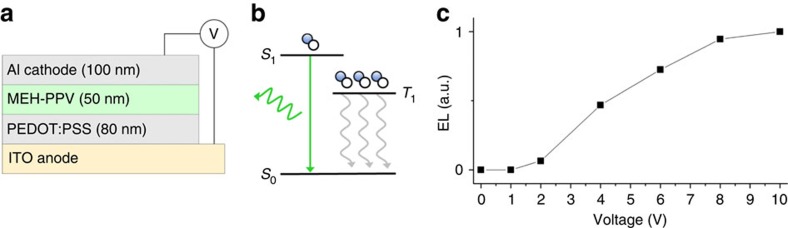
Device architecture and performance. (**a**) The OLED device, shown schematically, consists of a polymer bilayer (PEDOT:PSS for hole injection with MEH-PPV on top for light emission) between two electrodes (ITO on bottom, Al on top) and between which voltage is applied. (**b**) Energy-level diagram of exciton formation in the polymer film. When the ground state *S*_o_ is stimulated electrically into an excited singlet state *S*_1_, spin statistics imply that uncorrelated electrons and holes pair to form three triplet (*T*_1_) states for every singlet state, but they relax thermally (grey arrows) leaving ∼25% of singlet excitons that emit light (green arrow). (**c**) Electroluminescent light emission intensity, normalized to the maximum intensity at the highest voltage, is plotted as a function of voltage across the device.

To demonstrate the proof-of-concept, we consider the system for fundamental studies: an MEH-PPV film (electroluminescent polymer) sandwiched between an electron injection layer of PSS:PEDOT and an electrode (Fig. 1), using the optical activity of MEH-PPV directly for imaging.

### Adapting STED to operating organic LEDs

**Figure 2:**
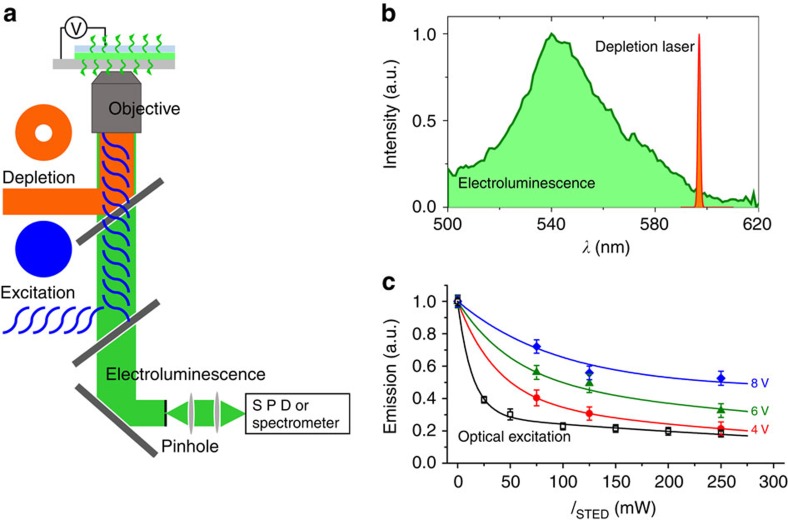
Experimental setup. (**a**) EL-STED microscope with wavelength-discriminated detection. Passing a current across an electroluminescent polymer field provides a wide-field excitation and the emission is passed through a pinhole in the image plane and detected on a single-photon diode or spectrometer. A ‘doughnut’ CW depletion beam is then focused to fill the field-of-view defined by the pinhole, quenching the majority of emissive excited states. The STED microscope can easily change excitation sources from optical to electrical, allowing PL and EL images to be taken sequentially. (**b**) Electroluminescence spectrum of MEH-PPV and the depletion laser, tuned to the red-side of the emission profile. This eliminates any absorption of the depletion beam by the sample while still allowing for efficient depletion. (**c**) Depletion characteristics for both optical STED (open squares) and EL-STED (closed symbols) as function of STED beam intensity. Error bars reflect fluctuations in the measured emission intensity, which was averaged over 10 s for each measurement. The depletion efficiency depends on the power of the depletion beam relative to the excitation density. As the brightness of the LED device is increased (increase of applied voltage), stimulated emission becomes less efficient to a point were depletion saturation is not obtainable with the depletion laser. The maximum power of the depletion beam corresponds to roughly 100 MW cm^−2^ on the sample.

Traditional STED imaging obtains sub-diffraction-limited images by optically modulating fluorescent probes between light and dark states using a combination of focused laser beams. The resulting emission is recorded from only a subset of the initially excited molecules. In the variant described here, molecules are initially excited by passing a current across the emissive polymer film to produce wide-field emission and a pinhole placed in the image plane reduces the field-of-view to roughly a 400 × 400-nm area, comparable to a confocal microscope (Fig. 2a). For photo-excitation, we use a pulsed, 800 nm laser that excites two-photon absorption of the probe molecules. To achieve super-resolution, we focus a doughnut-shaped continuous wave (CW) depletion beam, shifted to the red of the electroluminescence, onto the field-of-view region to selectively quench the majority of emissive states (Fig. 2b), then we scan the sample across the pinhole to collect an image. A subtle difference between photoluminescence (PL) and electroluminescence (EL) explains why the EL spectrum is blue-shifted from the PL spectrum. PL arises from photo-generated electron–hole pairs, which remain bound before recombining and therefore remains relatively low in energy, but EL arises from electrons and holes captured within the film that tend to be more loosely bound before recombination. We have followed a general practice in selecting a depletion beam that is red-shifted to the tail end of the emission spectrum of the dye being studied. Here we used a dichroic with a constant ∼10% reflection across the spectral region studied. The spectrum of the depletion beam was measured to remove the traces of it from the spectral imaging.

Triplet formation in electrical injection has long limited the maximum efficiency of organic LED devices because most excitations are immediately lost to triplet states that relax thermally and hence do not contribute to the device brightness. An interesting subtlety explains that this is not the case in the present application as one would expect triplets to typically interfere with the STED process, this is only for radiative triplets. Although 75% of excitations formed in OLEDs are triplet excitations^25^,^26^, their dominant mode of relaxation is thermal and thus do not emit, preventing the unfavourable spin statistics from interfering with the STED process^27^.

Although the emitted light in this study relies on no fluorescent labels, the spatial resolution is the same as when light emits alternatively in the traditional mechanism from optical stimulation: it depends on the capability to saturate depletion of emissive excited states. For the polymer films studied here, depletion as a function of depletion beam intensity is illustrated in Fig. 2c. The data are normalized to unity at the origin of no STED depletion (as absolute numbers depend greatly on details of the optical setup). For the conventional STED case of optical excitation, over 80% quenching can be achieved with roughly 100 mW (∼40 MW cm^−2^). For our present case of electrically generated excitons, at 6 and 8 V the electroluminescence is too intense to be depleted with our set-up, but at 4 V there is 80% depletion with a STED beam intensity of 250 mW (∼100 MW cm^−2^). While the curves seemingly have different shapes, we would anticipate that normalizing the *x*-axis (the STED intensity) by the saturation intensity of the chromophore would cause the curves to collapse onto a master curve, but no quantitative explanation of the shape differences is offered at this time. The main point is pragmatic: increasing the stimulation voltage actually reduces the STED spatial resolution.

### Imaging an operating organic LED

**Figure 3:**
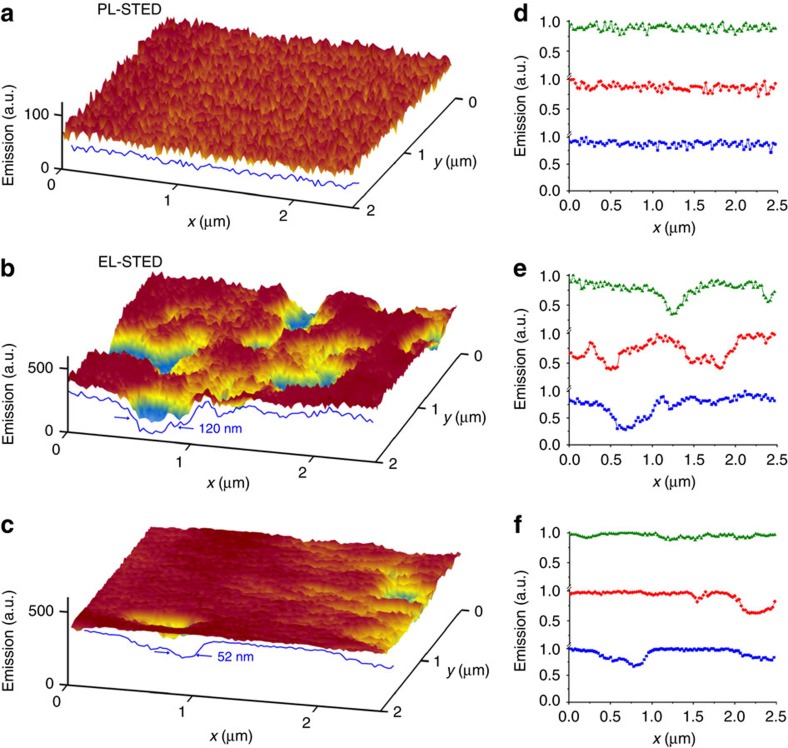
Images of a representative device. Pixel size is 25 nm. (**a**) Conventional STED imaging at voltage *V*=0 shows a homogeneous MEH-PPV film. (**b**) This is confirmed by representative line scans showing emission intensity plotted against position quantitatively. (**c**) The same region but with *V*=4 V. Electroluminescence is the light source. One sees regions of dim emission with dimensions ranging from 50 to 150 nm. (**d**) Line scans across the image quantify the sizes and shapes of the defects. (**e**) Image taken after annealing with ethanol to improve contact efficiency. One sees the defects are fewer but the remaining ones are uniform. (**f**) Representative line scans quantify post-annealed film quality. We have confirmed these patterns of behaviour in measurements of dozens of devices.

For benchmark perspective, first we show traditional optical STED images when the device was not operating, taking care to do so in the same spot as when the device was later put into operation. An optical excitation beam drives fluorescence emission from the MEH-PPV polymer that comprises the emissive layer of the OLED. Figure 3a illustrates this with representative cross-sections shown in Fig. 3b. The image shows, within the sensitivity and ∼50 nm spatial resolution, that the polymer film is uniform with good surface coverage. The same region was then imaged with EL-STED (Fig. 3c), revealing nanoscale features (bright and dim regions of various sizes) that are not observed in the traditional image. Regions of dim emission range, length 50–150 nm, are up to 50% less bright than other regions. Figure 3d shows representative cross-sections of this image. As the working and turned-off device were imaged in the same spot, one can be sure that the defect regions are not due to poor surface coverage, but instead arise from properties of the device.

Side-chain aggregation cannot explain this data, because though it could in principle contribute to heterogeneity it would occur at scales less than our spatial resolution of 50 nm. The contribution of polymer processing during device construction was explored by preparing a similar LED device but with a treatment known to increase the hole injection frequency, namely post-deposition treatment by ethanol dripping to improve contact quality at the PEDOT:PSS interface. The absolute bulk brightness increased in both bright and dim regions. More interestingly, relative to the untreated device, this produced fewer dim spots and brighter bright spots, as illustrated in the EL-STED image shown in Fig. 3e with line scans of the image shown in Fig. 3f. The surviving dim regions probably reflect extended domains of relatively poor-chain-packing density, which limit local conductivity and facilitate thermal relaxation of excitons due to higher chain mobility^20^,^21^,^28^. It is physically reasonable that charge transport would be more efficient among tightly packed chains, as chain-to-chain hopping is the rate-limiting step of charge transport in semiconducting polymer films^21^,^28^. Unfavourable chain conformations in extended regions of spin-cast films has long limited the efficiency of organic LEDs, as well as solar cell devices^25^,^29^,^30^, so a direct measure of how device performance relates to chain packing at the nanoscale can provide productive new insights into these devices.

### Wavelength-discriminated imaging

Next we attached a spectrometer and resolved the full-emission spectrum, exploiting the sensitivity of this polymer’s emission spectrum and photophysics to the polymer chain environment (packing density and aggregation, especially)^31^,^32^,^33^. Wavelength-discriminated images were constructed by measuring the PL emission spectrum using STED excitation, pixel-by-pixel, over large areas 640 × 800 nm (17 by 21 pixels).

**Figure 4:**
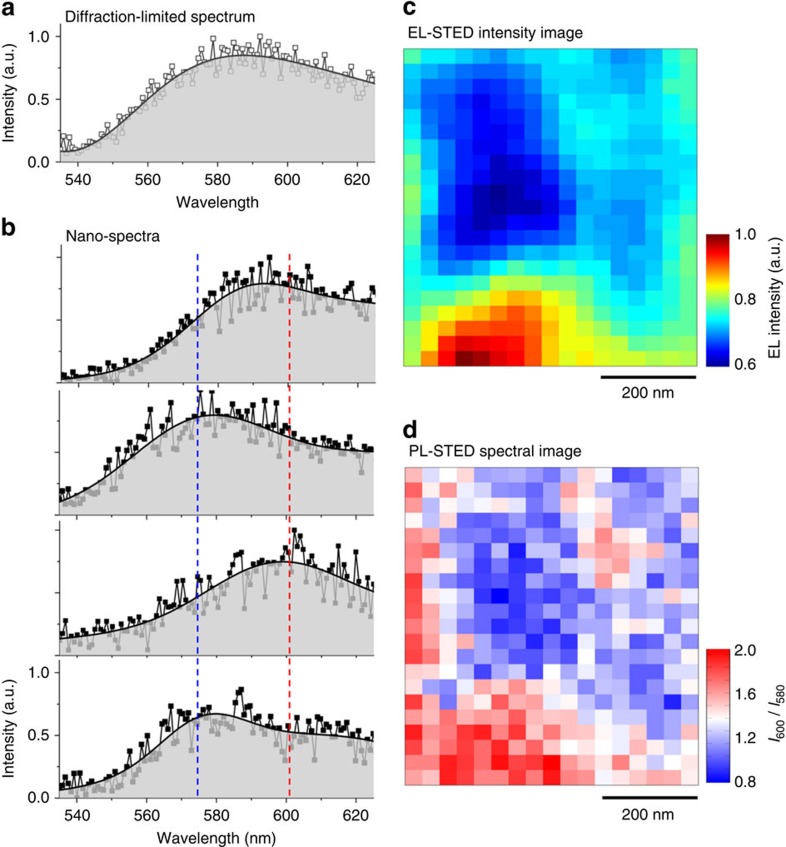
Wavelength-discriminated images. (**a**) A conventional photoluminescence measurement (intensity plotted against wavelength with diffraction-limited optical resolution) displays no dependence on location. (**b**) Photoluminescence spectra of the same working device taken with 50 nm spatial resolution depend on location, giving physical insight because red shift reflects domains where chains pack more densely. For quantification, we consider the ratio of luminescence intensity in the blue at 600 nm, to that in the red at 580 nm, *I*_600 nm_*/I*_580 nm_. (**c**) Super-resolution image of a small defect in an operating OLED device. Blue and red are low and high intensity, respectively. (**d**) Same region in the same LED imaged with PL-STED-spectral imaging. Blue and red are believed to be regions of relatively less dense- and more dense-packed chains, probably reflecting varying degrees of annealing during device preparation. Note the high correlation between bright emission and dense packing. The image correlation between **c** and **d** is 0.7.

What is the advantage relative to conventional imaging? A vivid illustration of what super-resolution brings to the table, relative to the conventional best diffraction-limited optical resolution of∼350 nm, comes when one compares the PL emission spectra of the MEH-PPV layer measured with the diffraction-limited excitation spot (Fig. 4a) to those collected with super-resolution (Fig. 4b). Unlike the diffraction-limited spectrum, spectra collected with∼50 nm resolution show changes in the spectral features depending on the location from which the spectrum was measured. To correlate these spectral changes to the EL process, we first image the EL of a region of the film with a prominent defect (EL-STED image, Fig. 4c). The same region was then imaged with PL spectral imaging, and a wavelength-discriminated image was constructed by analysing the ratio of emission intensities at wavelengths of 600 and 580 nm as the spectrum of this polymer red-shifts due to the densely packed chains (Fig. 4d). By comparing the two images (Fig. 4c,d) one finds a strong correlation between brightness and spectral features.

### Control experiments

Significant differences were confirmed in the wavelength-discriminated photoluminescence spectra of MEH-PPV in toluene solution, in films that had not been solvent-annealed and in films that had been thermally annealed (Supplementary Fig. 1). Also, we confirmed that features of the wavelength-discriminated spectra did not change when depletion was performed. This is illustrated in comparison to the fluorescence emission spectrum of a common dye, coumarin 153 in toluene, with (triangles) and without (squares) concurrent STED measurement. Each emission wavelength was depleted with the same efficiency (Supplementary Fig. 2).

## Discussion

A natural interpretation of this data is to conclude that thermal annealing is not democratic. Not affecting all spots equally, it leaves portions of the film incompletely annealed and therefore performing relatively poorly. Because the annealing process is known to cause red-shift of the MEH-PPV photoluminescence spectrum^34^, by this argument the poorly performing regions would correlate with regions showing blue-shifted spectra. Further interpreting the red-shift as arising from interchain interactions and efficient packing, the data suggest that local electroluminescence is primarily sensitive to changes in charge transport efficiency, which is higher in more tightly packed regions due to morphological heterogeneity^20^. EL is generally understood to be governed by a trade-off between charge transport, which is most efficient through tightly packed chains and quantum efficiency, which is highest in isolated chains due to fewer quenching mechanisms. From inspecting the data, we suspect that the observed bright regions have general features of tight packing and high conductance on mesoscopic length scales, with local defects on nanoscopic length scales. Our measurements would resolve the mesoscopic features, while not fully resolving defects on smaller length scales. This interpretation is guided by work using near-field scanning optical microscopy to image morphological heterogeneities in OLED films, where chain morphology was correlated with spectral features of MEH-PPV using single-chain spectroscopy, although not in working devices^15^,^20^. These techniques are also subject to the limitation of this technique that the observations are restricted to the surface whereas light is largely generated in the interior of the active layer^35^. Looking to the potential of this method in the future to enable other materials studies, it is pleasing to note that this study circumvented the uncertainty of knowing the complex role that chain morphology plays in carrier dynamics by imaging electroluminescence directly, using this generalizable method.

The perspective is this: while it is known that in conducting polymers there is trade-off between high-mobility regions to facilitate charge injection and low-mobility regions to promote recombination and emission, how best to manipulate molecular organization to maximize device efficiency based on this knowledge remains challenging^20^,^22^,^23^ given the huge range of possible processing parameters. Here we use STED microscopy techniques to perform tandem measurements of film morphology and local EL generation in operating OLEDs, finding that local emission brightness depends strongly on the nanoscale chain packing. These alternative characterization methods, based on the new ideas presented here, make a more direct connection between chain morphology and device function by imaging devices during routine operation. The methods described here were applied to a system selected for its interest for fundamental polymer OLED physics and also its historical place in OLED research. The ideas and methods are anticipated to extend naturally to systems in present-day OLED displays.

## Methods

### OLED fabrication

ITO-coated glass slides were sonicated in isopropyl alcohol for 1 h, then dried thoroughly. Conductive grade PEDOT:PSS (Sigma Aldrich) was shaken (3–4% in H_2_O) for 24 h, then solution-cast onto the ITO glass at 1,500 r.p.m. for 2 min. The film was annealed for 2 h at 180 °C under vacuum. A solution of MEH-PPV (Sigma Aldrich) in toluene (∼5 mg ml^−1^) was stirred for 24 h then filtered through a 1-μm filter. A film was then solution-cast onto the PEDOT:PSS film at 1,500 r.p.m. for 2 min. The film was then dried at 80 °C for 30 min. Finally, 40 nm of aluminium was thermally evaporated onto the OLED through a shadow mask, which defined active regions roughly 1 × 1 mm. The active regions where connected to a voltage supply.

For the ethanol-annealing step of the device presented in Fig. 2, everything was the same except that after the spin-coating and annealing of the PEDOT:PSS layer, the entire film was dipped into ethanol, quickly removed and dried at 80 °C for 30 min. This increases the conductivity of the PEDOT:PSS layer and can also improve the surface roughness. From this treatment, we expect not only better hole injection but also better contact quality between PEDOT:PSS and MEH-PPV layers.

### STED set-up

Optical excitation is from an 800 nm pulsed femtosecond laser (Spectra Physics Mai Tai). Depletion is from a CW diode laser centred at 592 nm (MPB Communications), passed through a custom-made vortex phase mask which gives the beam a doughnut shape at the sample. A 20-μm pinhole placed in the image plane of the microscope reduces the field-of-view to the point that the depletion beam fills the image region defined by the pinhole. Thus, depletion of this region can be fully accomplished. Note while many implementations of STED use pulsed excitation and depletion lasers, the depletion in EL-STED must be carried out with a CW depletion laser because the voltage supply used for electrical excitation provides a continuous excitation. Detection is from a single-photon diode. An offset is subtracted that comes from reflection of the depletion beam from solid interfaces in the system.

### Wavelength-discriminated STED

The system is the same except for the detection step. The photoluminescence is dispersed off a grating (300 grooves per mm, Thor Labs) and detected on an emCCD camera (Hamamatsu). The entire spectrum of emission was recorded at each point in the image, pixel-by-pixel.

STED-spectral imaging requires that depletion does not alter the spectral features by, for instance, depleting certain wavelengths more efficiently than others. Because the depletion beam comes from a narrowband CW laser, it does not uniformly cover the entire emission spectrum of the fluorescent probe. To ensure that the spectral features are not altered by depletion, we record the emission spectrum of coumarin 153 (Sigma Aldrich) dissolved in toluene (Sigma Aldrich) with and without the STED beam (Supplementary Fig. 2). While the overall intensity of the spectrum is greatly reduced, the spectral features remain unchanged by the depletion beam. This is even more obvious when the spectra are normalized (Supplementary Fig. 2, inset), where a residual gives a flat line.

### Data collection

*EL-STED imaging*. Images were collected over 2.5 × 2-μm regions of the sample. The sample was scanned with a step size of 20 nm. Each image took roughly 15 s to collect. Note the time resolution of the instrument is limited by the scanning stage, not the signal detection. The signal was detected on a single-photon detector (ID Quantique).

*STED-spectroscopy*. The region imaged with STED-spectroscopy was roughly 600 × 800 nm. Individual spectra were integrated for 50 ms with a 40-nm step size. The data were then analysed with home-written programs.

### Data availability

The authors declare that the data supporting the findings of this study are available within the article and its Supplementary Information Files.

## Additional information

**How to cite this article:** King, J. T. & Granick, S. Operating organic light-emitting diodes imaged by super-resolution spectroscopy. *Nat. Commun.* 7:11691 doi: 10.1038/ncomms11691 (2016).

## Supplementary Material

Supplementary Information Supplementary Figures 1-2
