# Supplementary material for: Operating organic light-emitting diodes imaged by super-resolution spectroscopy
Source: Nat Commun. 2016 Jun 21;7:11691. doi: 10.1038/ncomms11691 (PMC5512612; doi:10.1038/ncomms11691)
Supplement: Supplementary Information — Supplementary Figures 1-2 [file ncomms11691-s1.pdf]

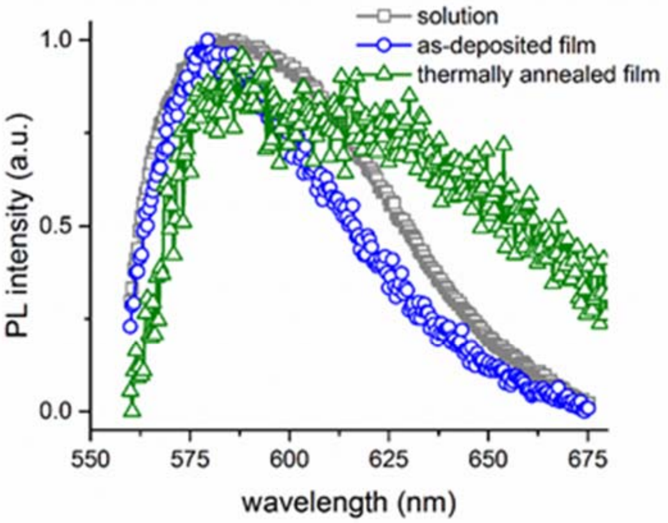

Supplementary Figure 1. Comparison of photoluminescence spectra of MEH-PPV in toluene solution, in films that have not been annealed, and in films that have been thermally annealed.

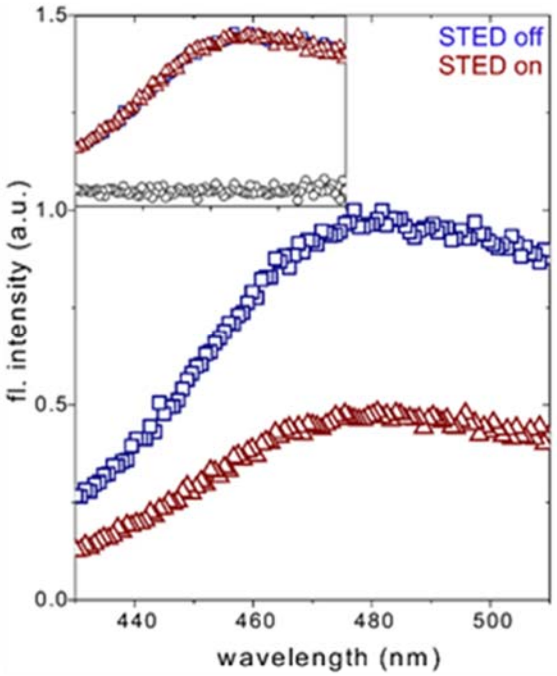

Supplementary Figure 2. Comparison of the fluorescence emission spectrum of a common dye, Coumarin 153 in toluene, with (triangles) and without (squares) concurrent STED measurement. The features of the spectrum do not change when depletion is performed, meaning that each emission wavelength is depleted with the same efficiency. (Inset) Ratio of the spectra is constant (gray circles).
